# Innate antiviral defense demonstrates high energetic efficiency in a bony fish

**DOI:** 10.1186/s12915-021-01069-2

**Published:** 2021-07-13

**Authors:** Mark P. Polinski, Yangfan Zhang, Phillip R. Morrison, Gary D. Marty, Colin J. Brauner, Anthony P. Farrell, Kyle A. Garver

**Affiliations:** 1 Fisheries and Oceans Canada Pacific Biological Station, 3190 Hammond Bay Road, Nanaimo, V9T6N7 Canada; 2 Faculty of Land and Food Systems, University of British Columbia, MCML 344-2357 Main Mall, Vancouver, V6T1Z4 Canada; 3 Department of Zoology, University of British Columbia, 6270 University Blvd, Vancouver, V6T1Z4 Canada; 4 Animal Health Centre, Ministry of Agriculture, Food and Fisheries, 1767 Angus Campbell Rd, Abbotsford, V3G2M3 Canada

**Keywords:** Salmon, Piscine orthoreovirus (PRV), Infectious hematopoietic necrosis virus (IHNV), Respiratory performance, Innate immunity, Acute stress

## Abstract

**Background:**

Viruses can impose energetic demands on organisms they infect, in part by hosts mounting resistance. Recognizing that oxygen uptake reliably indicates steady-state energy consumption in all vertebrates, we comprehensively evaluated oxygen uptake and select transcriptomic messaging in sockeye salmon challenged with either a virulent rhabdovirus (IHNV) or a low-virulent reovirus (PRV). We tested three hypotheses relating to the energetic costs of viral resistance and tolerance in this vertebrate system: (1) mounting resistance incurs a metabolic cost or limitation, (2) induction of the innate antiviral interferon system compromises homeostasis, and (3) antiviral defenses are weakened by acute stress.

**Results:**

IHNV infections either produced mortality within 1–4 weeks or the survivors cleared infections within 1–9 weeks. Transcription of three interferon-stimulated genes (ISGs) was strongly correlated with IHNV load but not respiratory performance. Instead, early IHNV resistance was associated with a mean 19% (95% CI = 7–31%; p = 0.003) reduction in standard metabolic rate. The stress of exhaustive exercise did not increase IHNV transcript loads, but elevated host inflammatory transcriptional signaling up to sevenfold. For PRV, sockeye tolerated high-load systemic PRV blood infections. ISG transcription was transiently induced at peak PRV loads without associated morbidity, microscopic lesions, or major changes in aerobic or anaerobic respiratory performance, but some individuals with high-load blood infections experienced a transient, minor reduction in hemoglobin concentration and increased duration of excess post-exercise oxygen consumption.

**Conclusions:**

Contrary to our first hypothesis, effective resistance against life-threatening rhabdovirus infections or tolerance to high-load reovirus infections incurred minimal metabolic costs to salmon. Even robust systemic activation of the interferon system did not levy an allostatic load sufficient to compromise host homeostasis or respiratory performance, rejecting our second hypothesis that this ancient innate vertebrate antiviral defense is itself energetically expensive. Lastly, an acute stress experienced during testing did not weaken host antiviral defenses sufficiently to promote viral replication; however, a possibility for disease intensification contingent upon underlying inflammation was indicated. These data cumulatively demonstrate that fundamental innate vertebrate defense strategies against potentially life-threatening viral exposure impose limited putative costs on concurrent aerobic or energetic demands of the organism.

**Supplementary Information:**

The online version contains supplementary material available at 10.1186/s12915-021-01069-2.

## Background

The evolution and maintenance of immune systems for the purpose of parasite resistance is critical for animal survival; yet, immunological competence necessitates an internal resource allocation that could otherwise be used toward growth, reproduction, or other activities. Thus, parasite resistance incurs a putative energetic cost against all fitness-enhancing processes. This concept of “trading-off” energetic demands between life-history traits in the face of dynamic ecological challenges is the foundation of life-history theory [1].

Considerable debate has surrounded exactly what the overall energetic cost of parasite resistance might entail. Some immune functions appear energetically expensive [2–4]. For example, humans mounting a fever response following typhoid vaccination transiently increase metabolic rate by 16% [5], while calorie-restricted lizards experience a near 50% reduction in ovarian follicle mass development when tasked with wound healing compared to unwounded individuals [6]. In contrast, other immune responses seem to have little to no energetic cost [7–10], e.g., increased allelic polymorphism of erythrocyte *Plasmodium*-target antigens that enhance malaria resistance [8, 11]. These varied responses have hindered our ability to accurately predict the likelihood for important tradeoffs and suggest that energetic costs of parasite resistance may be pathway- or even parasite-specific.

Defense against viruses in eukaryotic vertebrates involves the coordination of both an innate and adaptive immune system, primarily under the regulation of interferon-sensitive pathways. Specifically, viral recognition by innate pattern recognition receptors triggers local transcriptional activation of interferons. Interferon in turn facilitates transcription of interferon-stimulated genes (ISGs) that enable local cells to mount antiviral molecular defenses, recruit immune cells to the site of infection, and restrain pro-inflammatory responses [12, 13]. Interferons also play a critical role in activating the adaptive responses of T- and B-cells to target the invading virus in order to curtail persistent infections and defend against re-exposure [13].

Despite extensive mapping of the interferon molecular cascade in vertebrate systems, the metabolic costs associated with these responses are largely unclear. Data do suggest that, at a cellular level, activation of interferon promotes glycolysis (an anaerobic pathway) and in some cases increases mitochondrial respiration (an aerobic pathway) [14]. However, in most instances, it is questionable as to whether these costs exceed the allostatic capacities of an organism to absorb minor perturbations that would either affect homeostasis [15] or impact long-term growth and reproduction of the organism [9, 10, 16].

Dynamic environmental conditions further complicate assessments of viral-associated fitness costs, especially in ectotherms where environmental conditions directly influence metabolism. Specifically, studies identifying physiologically relevant costs for parasite resistance in vertebrate systems have mostly been conducted under resource-limiting conditions untenable to maintain metabolic homeostasis independent of immunological functioning [9, 17]. Thus, although these studies provide evidence for prioritizing pathways during extensive resource limitation, there is currently little data to indicate the quantity of energetic resources required to fully engage an antiviral immune response (i.e., the energy required for uncompromised resistance).

In this study, we set out to test three hypotheses relating to the metabolic and fitness costs of innate antiviral resistance and tolerance in a vertebrate model, the sockeye salmon (*Oncorhynchus nerka*), a teleost fish. First, we hypothesized that under stable environmental conditions conducive for maintaining metabolic homeostasis, virus resistance (defined here as actions specifically taken to curb virus colonization and replication) incurs a higher energetic cost relative to virus tolerance (allowing virus colonization and replication to progress relatively unimpeded) or non-exposure. This follows the logic that tolerance would not occur if energetic costs for active resistance were lower than the costs attributed to viral parasitism, which has been recently estimated to be minimal—approximately 1% of a eukaryotic cell’s energy budget [18]. Second, we hypothesized that at least part of the energetic costs for viral resistance would come from the induction of the interferon response system and that activation of an interferon response adequate to protect an organism from a life-threatening viral infection would incur sufficient energetic costs to impact metabolic homeostasis. Here, we assume that the metabolic costs previously identified in association with the induction of interferon pathways at a cellular level [14] translate more broadly to tissues and organs. Lastly, we hypothesized that the re-allocation of resources in association with an acute stress event (i.e., an exhaustive chase followed by recovery in isolated confinement) impairs immunological functions, increases viral infection (i.e., viral load), and intensifies disease (i.e., impairs tissue/organ structure and functions). This follows the rationale that the energy required for an antiviral immune response is likely to be traded off in order to maximize energy availability for immediate flight responses and that this resource re-allocation hinders host resistance for minimizing viral replication and disease [19, 20].

The putative metabolic and fitness costs of viral infection were identified in our study by evaluating the physiological and immunological responses of sockeye salmon at three strategic time points (1, 4, and 9 weeks post-challenge) following intraperitoneal virus injections. We independently tested both a low-virulence reovirus (Piscine orthoreovirus genotype-1; PRV) and a high-virulence rhabdovirus (infectious hematopoietic necrosis virus; IHNV). PRV (specifically the PRV-1 genotype found in western North America) is not known to cause disease in sockeye salmon even under circumstances of persistent high-load systemic infection, host saltwater adaptation, or viral co-infection, and even though the virus targets erythrocytes as its primary replication site [21, 22]. Nevertheless, the PRV-1 variant used in this study may have virulence potential under some circumstances because it has been associated with mild heart inflammation [23] and suggested as a putative contributing factor to jaundice syndrome [24] in other salmon species. In contrast, IHNV causes significant disease (infectious hematopoietic necrosis; IHN) and mortality in sockeye salmon by targeting the endothelial cells of blood capillaries and hematopoietic tissues of the kidney. The virus has been one of the most impactful pathogens to salmon aquaculture over the last century [25, 26], and salmon (including sockeye) are known to employ a number of IHNV-resistance strategies that include the robust induction of interferons [25, 27].

Given that respiration is a critical life support system for all vertebrates and (by providing an adequate oxygen supply) acts as a primary regulatory mechanism for energy consumption, any metabolic costs sufficient to surpass the allostatic capacity of an organism to maintain homeostasis should become readily evident through changes in respiratory performance. Here, we comprehensively assessed respiratory phenotype using a recently developed integrated respiratory assessment protocol (IRAP) that measures a fish’s respiratory capabilities and capacities using 15 indices [15, 28–30]. Specifically, standard metabolic rate (SMR) and maximum oxygen uptake (ṀO2_max_) directly measure aerobic needs and capabilities, respectively. Absolute aerobic scope (AAS = ṀO2_max_ − SMR) and factorial aerobic scope (FAS = ṀO2_max_/SMR) can then be calculated to assess aerobic capacity. Excess post-exercise oxygen consumption (EPOC) directly measures a fish’s capability to recover from exhaustion, while EPOC duration (EPOC_dur_) identifies the time required for this recovery. Routine metabolic rate (RMR) and daily energy expenditure (DEE) quantify the energy spent for living in the laboratory. Time spent above 50% maximum oxygen uptake rate (T_0.5ṀO2max_) and time spent above 80% maximum oxygen uptake rate (T_0.8ṀO2max_) assess an individual fish’s behavioral activity. Lastly, critical oxygen level (O_2crit_) and incipient lethal oxygen saturation (ILOS) directly assesses hypoxia tolerance, which can then be used to calculate a fish’s scope for oxygen deficit (SOD), the factorial scope for oxygen deficit (FSOD), and accumulated oxygen deficit (AOD) to assess anaerobic capabilities [28]. For our study, these respiratory measurements were complimented by a thorough assessment of hemoglobin status [specifically by measuring hematocrit (Hct) and hemoglobin (Hb) concentration] and by assessing oxygen binding capabilities [Hb saturation at 21% O_2_ (Hb_21%_), plasma pH, and the partial pressure for 50% O_2_ saturation (P_50_)] in combination with monitoring of immune gene transcriptional signaling and viral transcriptional loads in organs targeted by PRV and IHNV (the blood and kidney), respectively.

## Results

### Sockeye salmon tolerated high-load PRV infections with only mild transient consequences to oxygen transport and exhaustive chase recovery

**Fig. 1:**
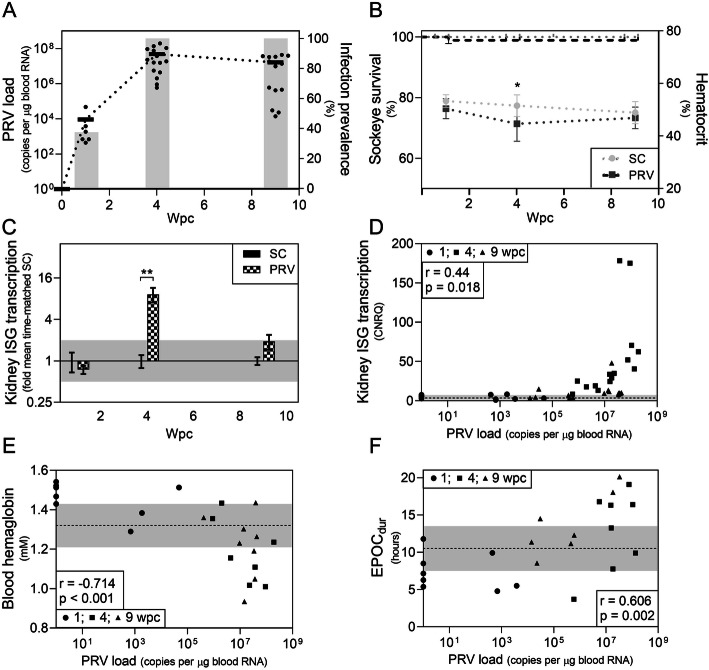
Response of sockeye salmon to PRV infection. **a** Mean (line) and individual (dot) PRV RNA loads and infection prevalence (bars) measured at 0, 1, 4, and 9 weeks post-challenge (wpc). **b** Kaplan-Meier survival curves (left axis) of saline control (SC) and PRV-injected (PRV) sockeye (log-rank p = 0.32) and mean (box) ± SD hematocrit (right axis) at 1, 4, and 9 wpc; *p < 0.05 by 2-way ANOVA and Dunnett’s multiple comparison tests of arcsine-transformed hematocrit values. **c** Mean fold change (± SE) of interferon-stimulated gene (ISG) transcription measured as mean *mx1*, *rsad2*, and *eif2ak2* mRNA transcripts relative to their expression in time-matched controls (SC); **p < 0.01 by 2-way ANOVA and Dunnett’s multiple comparison tests of corrected normalized relative quantities (CNRQ); minimum twofold change suggestive of biological relevance is shaded. **d** Correlation of ISG transcript abundance relative to log-PRV RNA load; Spearman r and associated p-value provided; mean (dotted line) ± SD (shaded) of SC ISG transcription suggestive of minimum threshold for biological relevance. **e** Correlation of blood hemoglobin concentration and **f** duration of excess post-exercise oxygen consumption (EPOC_dur_) relative to log-PRV RNA load; Spearman r and associated p-value provided; mean (dotted line) ± SD (shaded) of SC is indicated in both instances as suggestive of minimum threshold for biological relevance

Intraperitoneal injection of PRV into salmon causes a well-characterized persistent, high-load systemic infection where peak viral transcriptional and protein loads are reached approximately 1 month following infection at 10–12°C [31]. PRV infection dynamics within sockeye salmon of our study were congruent with these previous findings (Fig. 1a). At 1 week post-challenge (wpc), 6 of 16 fish (38%) had developed a moderate systemic blood infection. At 4 wpc PRV infection was detected in the blood of 15 of 16 sampled fish with a mean (± SE) blood load of 4.9 (± 1.5) × 10^7^ reverse-transcribed L1 genome segment copies per μg total blood RNA—equivalent to approximately 1.2 ± 0.4 × 10^10^ copies per mL blood. One fish had a negative blood result but kidney tested positive at 5.4 x 10^6^ copies per μg total kidney RNA thus we consider this fish to have been systemically infected. At 9 wpc, PRV infection prevalence remained at 100% and mean infection intensity remained high (1.6 ± 0.4 × 10^7^ copies per μg blood RNA or approximately 3.5 ± 1.0 × 10^9^ copies per mL) in 15 of 16 fish, and 1.9 x 10^5^ copies per μg total kidney RNA was detected in the 16th fish.

PRV infections were not associated with any mortality, morbidity, or loss of body condition (Fig. 1b; Additional file 1: Fig. S1). No lesions in PRV-infected fish were suggestive of morbidity (Additional file 2) and PRV did not cause either an increase in prevalence or severity of pathology in the heart, skeletal muscle, kidney, liver, or spleen of sockeye salmon relative to controls (Additional file 1: Table S1), despite its putative association with mild to moderate disease in other salmon species [31].

High-load PRV infection did produce an interferon response, increasing mean ISG expression by approximately tenfold at peak infection (4 wpc) as measured by the equal-weighted mean of *mx1*, *rsad2*, and *eif2ak2* mRNA transcripts which code for three prominent interferon-stimulated proteins: Myxovirus resistance (Mx1), Viperin, and RNA-activated protein kinase (PKR), respectively (Fig. 1c). Furthermore, systemic PRV load was positively correlated with ISG expression in the kidney (Fig. 1d). Expression of *il1b* transcripts that code for the inflammatory cytokine interleukin-1β were not significantly elevated in the kidney of PRV-infected fish at any assessed time points in this study (Additional file 1: Fig. S2) nor was *il1b* expression correlated with PRV load (Fig. S3). Cumulatively, these data indicated a moderate systemic host recognition of PRV which occurred during peak infections but was not associated with increased inflammatory signaling and/or disease.

Respiratory performance of sockeye salmon was largely uncompromised as a result of PRV infection. Notably, 13 of the 15 respiratory indices were unchanged relative to time-matched control fish. However, two putative respiratory changes were observed. High variability and elevated (mean 64%; 95% CI = 21-107%; *p* < 0.002) EPOC was noted within SC fish at 1 WPC relative to both PRV and IHN. The duration of excess post-exercise oxygen consumption (EPOC_dur_) following an exhaustive chase was also prolonged by 43% (95% CI = 0.1–85%; p = 0.05) at the early PRV persistence phase (9 wpc; see Additional file 1: Fig. S1) without any significant effect on ṀO2_max_ or total EPOC.

In terms of red blood cell function, PRV infection did not reduce either the maximum oxygen-carrying capacity of the blood as measured by hemoglobin (Hb) concentration and Hb saturation at 21% oxygen or the partial pressure for 50% oxygen saturation of Hb (P_50_) relative to controls at any time point during the 9-week study (p > 0.12 for all time-matched comparisons, Additional file 1: Fig. S4). Hematocrit (Hct) was not affected in PRV-infected fish relative to controls (overall mean 43 ± 2% SC vs mean 41 ± 2% PRV; p > 0.27) when measured in IRAP fish after the extreme hypoxia treatment—a treatment expected to manifest lower and more steady-state Hct than when sampled directly from dip-netted fish (Additional file 1: Fig. S4). However, mean Hct was temporarily reduced (i.e., only at 4 wpc during the peak of PRV infection; Fig. 1b) by approximately 8% (95% CI = 1–15%; p = 0.02) in non-IRAP fish that were batch-sampled directly from their holding aquaria.

PRV load was negatively correlated with Hct and Hb concentration in non-IRAP fish and positively correlated with EPOC_dur_ in IRAP fish (Fig. 1e, f). Hb concentration was clearly compromised (beyond one standard deviation of the mean SC Hb value) in most individuals with more than approximately 5 × 10^6^ PRV L1 copies per μg total blood RNA (roughly 10^9^ copies per mL). While these relationships were independent of sampling time, they were clearly driven by individuals with the highest infections, but even then, only approximately 50% of individuals had an atypical Hb concentration or EPOC_dur_ at any given time. Therefore, although some high-load PRV-infected fish showed a minor reduction in oxygen transport capacity and/or required more time to recover from oxygen debt, these effects appeared contingent on both high-load PRV infections as well as individual-specific factors unidentified in this study.

### Reduction of standard metabolic rate occurred as part of effective early resistance against potentially lethal IHNV exposure

IHNV can cause a life-threatening disease (IHN) in sockeye salmon, but disease occurrence and severity are highly variable among individuals [27] and also are dependent on external factors such as host life stage, viral genotype, and the environment [25]. Our study was designed to induce 20–30% mortality of sockeye salmon smolts following an intraperitoneal injection challenge with expected peak infection loads occurring at approximately 1 wpc. This was based on previously established IHNV challenge kinetics [22, 32] and pilot challenges in our facility involving injections of serial dilutions of IHNV (Additional file 1: Fig. S6).

**Fig. 2:**
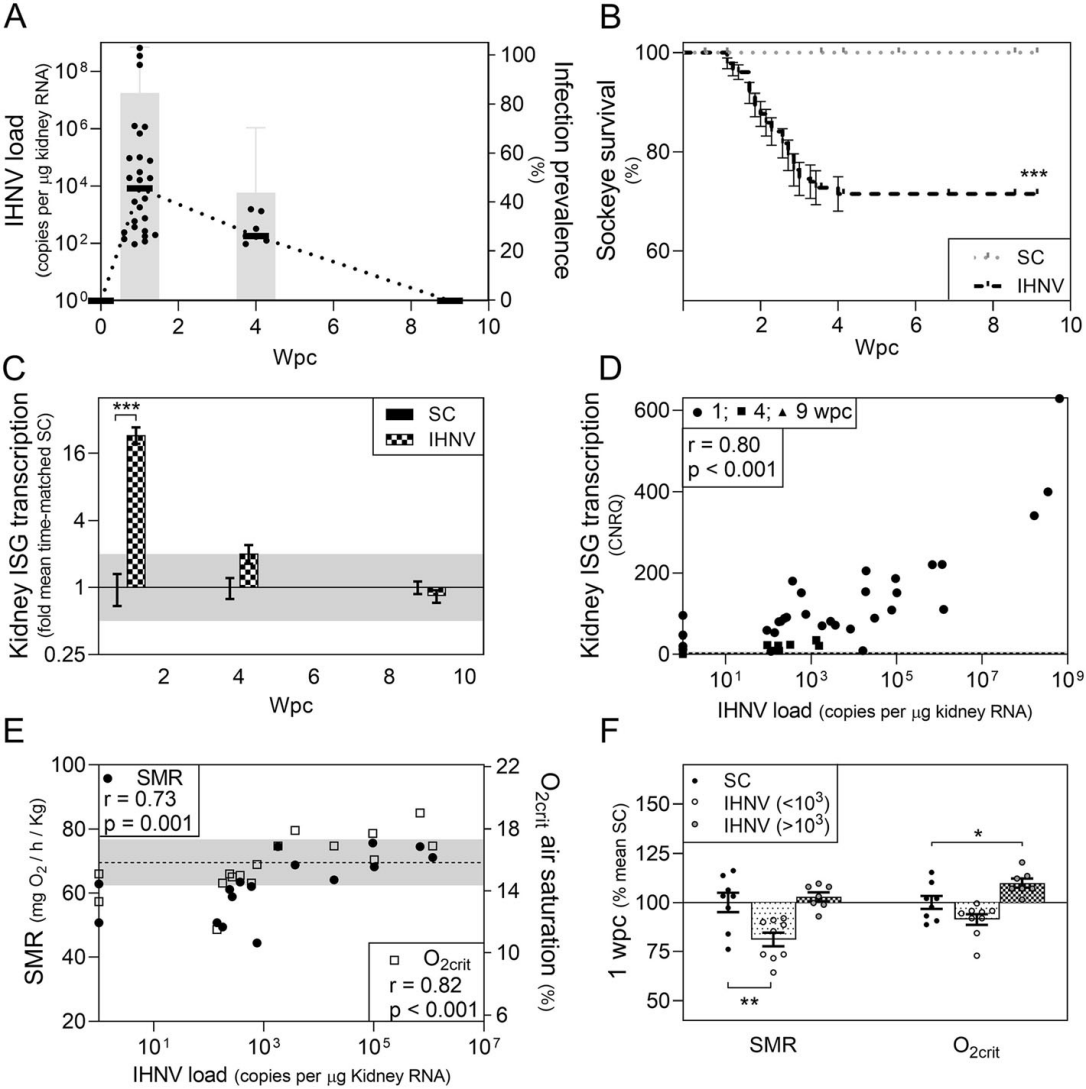
Response of sockeye salmon to IHNV infection. **a** Mean (line) and individual (dot) IHNV transcript loads and infection prevalence (bars) measured at 0, 1, 4, and 9 weeks post-challenge (wpc). **b** Kaplan-Meier survival curves of saline control (SC) and IHNV-injected (IHNV) fish (log-rank p < 0.001) ± SE. **c** Mean fold change (± SE) of interferon-stimulated gene (ISG) transcription measured as *mx1*, *rsad2*, and *eif2ak2* mRNA transcripts relative to their mean expression in time-matched controls (SC); ***p < 0.001 by 2-way ANOVA with Dunnett’s multiple comparison tests using corrected normalized relative quantities (CNRQ); minimum twofold change suggestive of biological relevance is shaded. **d** Correlation of ISG transcript abundance relative to log-IHNV transcriptional load; Spearman r and correlative p-value provided; mean (dotted line) ± SD (shaded) of SC ISG transcription suggestive of minimum threshold for biological relevance. **e** Correlation of standard metabolic rate to log-IHNV transcriptional load; Spearman r and associated p-value provided; mean (dotted line) ± SD (shaded) of SC fish suggestive of minimum threshold for biological relevance. **f** Mean ± SE (bar) and individual values (dots) for standard metabolic rate (SMR) and critical oxygen threshold (O_2crit_) of IHNV-resistant (transcript loads < 10^3^/μg) and IHNV-susceptible (transcript loads > 10^3^/μg) fish relative to SC; *p < 0.05, **p < 0.01 by 1-way ANOVA and Dunnett’s multiple comparison tests

As anticipated, IHNV load and prevalence peaked in hematopoietic tissues of salmon at approximately 1 wpc and resistance to IHNV dissemination and replication was highly variable between individuals consistent with previous observations [27, 33] (Fig. 2a). Furthermore, mortality associated with IHNV began at 1 wpc and continued through 4 wpc resulting in a cumulative mortality of 29% (Fig. 2b). Of the 32 individuals screened at peak infection (1 wpc), three fish had high-load kidney infections >10^8^ IHNV genome copies per μg kidney RNA that corresponded with severe interstitial cell necrosis and mild to severe renal tubular necrosis characteristic of an IHN disease state (Additional file 2). Twenty-four fish had mild to moderate infection loads within the kidney between 10^2^ and 10^6^ IHNV genome copies per μg total RNA. These infected fish did not have lesions indicative of morbidity, but they did have an elevated prevalence of mild interstitial hematopoietic cell hyperplasia suggestive of an increased demand for erythrocytes or white blood cells somewhere in the body (Additional file 1: Table S1; Additional file 2). The remaining five individuals resisted IHNV dissemination and/or systemically cleared the virus to below detectable levels by 1 wpc with no evidence for virus-associated lesions. The IHNV load in all surviving fish at 4 wpc was reduced to <2 × 10^3^ copies per μg RNA in the kidneys; by 9 wpc, IHNV could not be detected in any survivors (Fig. 2a). These data support previous indications that primary IHNV resistance is determined early during the infection processes [27] and susceptible individuals either succumb to disease or resistant individuals are able to quickly clear the infection in most instances without developing long-term carrier status [32].

Correspondingly, mean transcriptional ISG expression revealed an interferon response that was highly correlated with IHNV genomic load and was upregulated in the kidney over a mean of twentyfold at 1 wpc peak infection but not during later stages of recovery (Fig. 2c, d). Thus, although not necessarily predictive of effective resistance, robust activation of ISGs by IHNV in a load-dependent manner was clearly evident [34]. In contrast, *il1b* transcriptional expression was elevated in the kidney of some IHNV-infected fish (Additional file 1: Fig. S2); however, this was not specifically correlated with IHNV load (Additional file 1: Fig. S3). Rather, *il1b* transcription appeared conditional on the combined effect of experiencing an IRAP assessment in addition to IHNV exposure (see next section), indicating that individual experiences also strongly affect inflammatory signaling during IHNV infection.

Both SMR and O_2crit_ (a measure calculated in part using SMR) were highly correlated with IHNV transcriptional kidney load and were suggested to be biologically relevant as evidenced by measures trending below and/or above one standard deviation of time-matched saline controls (SC) (Fig. 2e). Furthermore, inflection points in both indices occurred at approximately 10^3^ IHNV transcript copies per μg RNA whereby all fish with peak IHNV loads >10^3^ had SMR above 64 mg/kg/h and a O_2crit_ above 16%, while all fish with peak IHNV loads <10^3^ had SMR and O_2crit_ below these values (Additional file 1: Fig. S5). Thus, the more IHNV-resistant fish (i.e., those able to keep peak IHNV loads <10^3^ copies/μg) showed a reduced SMR relative to controls by a mean of 19% (95% CI = 7–31%; p = 0.003) and an uncompromised, if not slightly reduced, O_2crit_ (mean reduction 9%; 95% CI = 0–18%; p = 0.07) relative to SC (Fig. 2f). In contrast, more susceptible fish (those unable to keep IHNV infections below 10^3^ copies/μg) had a similar SMR but compromised (higher) O_2crit_ (mean increase 10%; 95% CI = 0–20%; p = 0.05) when compared with controls. These data suggest that early IHNV resistance in sockeye involves a reduction in SMR, which putatively protects against environmental hypoxia while fighting systemic infections.

Remaining IRAP indices were not meaningfully impacted by either IHNV exposure or infection, except that AOD became highly variable in IHNV-exposed fish during peak infection and appeared to be compromised in at least 2 individuals (Additional file 1: Fig. S1). Assessments relating to blood oxygen-carrying capacity identified a mean 8% reduction (95% CI = 3–13%; p = 0.001) of Hct in IHNV-exposed fish at 1 wpc irrespective of viral load (Fig. S4). Interestingly, this was not reflected in post-IRAP Hct values. Instead, post-IRAP Hct values were reduced later at 4 and 9 wpc in survivors at a time when Hct in non-IRAP cohort fish had returned to approximate SC values (Additional file 1: Fig. S4). We hypothesize that these changes may be due to IHNV-associated reductions in stress hormone-induced red blood cell swelling between IRAP and post-IRAP fish although this was not verified.

### IRAP assessments altered transcription of antiviral (*ifna*) and inflammatory (*il1b*) cytokines but not ISG transcription or viral replication kinetics

Each IRAP assessment began with an exhaustive chasing protocol followed by 3.5 days of undisturbed assessments and concluded with a severe, acute anaerobic challenge (see the “Methods” section for full description) from which fish could be revived. The exhaustive chase is known to cause a severe metabolic and acid-base disturbance, and an endocrine (notably cortisol and catecholamine) “fright” response [reviewed by 29, 35], which is enhanced by a standardized post-chase air exposure [35]. Consequently, fish sampled at the end of an IRAP (4 days following the exhaustive chase event) provided a means to identify how acute stress might impact the progression of virus replication and host antiviral transcriptional signaling of infected fish.

**Fig. 3:**
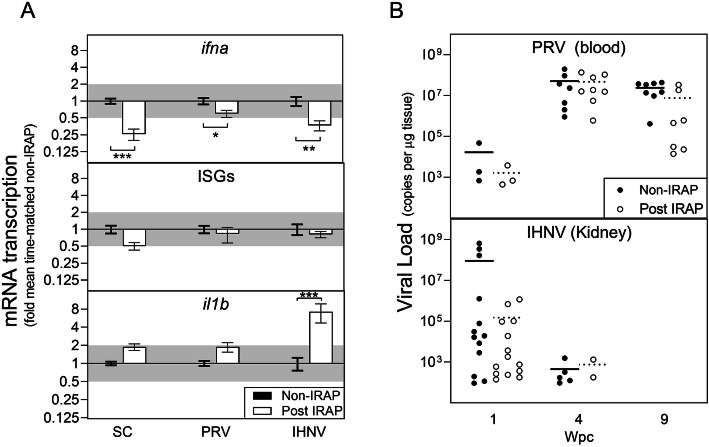
Effects of acute stress (IRAP-associated exhaustive chase and isolated confinement) on sockeye salmon interferon responses and PRV/IHNV load. **a** Mean (± SE) mRNA transcripts coding for type-1 interferon (*ifna*), three prominent interferon-stimulated genes (ISGs; myxovirus resistance, viperin, and protein kinase-R), and interleukin-1β (*il1b*) presented as fold change time-matched mean expression of non-IRAP (tank) averaged across multiple time points relative to their treatment-specific means; saline control (SC), PRV-injected (PRV), IHNV-injected (IHNV); *p < 0.05, **p < 0.01, ***p < 0.001 by 1-way ANOVA with Dunnett’s multiple comparison tests using corrected normalized relative quantities (CNRQ); minimum twofold change suggestive of biological relevance is shaded. **b** Mean (line) and individual (dot) RNA loads of PRV in the peripheral blood or IHNV in the anterior kidney measured at 0, 1, 4, or 9 weeks post-challenge (wpc)

We found that fish sampled at the end of the IRAP assessment had reduced *ifna* transcription relative to non-IRAP fish irrespective of treatment, with the greatest impact in SC fish (Fig. 3a). Interestingly, the reduction in *ifna* transcription did not notably affect ISG transcription, suggesting that alternative transcriptional promoters to IFNa were also at play with regard to both IHNV and PRV recognition and ISG induction; we suspect type-3 interferon (*ifng*) played a substantial role [27], although not confirmed in this study.

Contrary to our initial hypothesis, IRAP did not exacerbate IHNV or PRV loads. Indeed, mean loads were nominally lower in most instances following IRAP relative to non-IRAP sampled fish (Fig. 3b). However, transcription of *il1b* was elevated by a mean of approximately sevenfold (95% CI three- to ninefold; p ≤ 0.0001) in IHNV-challenged fish following IRAP but was not altered in either SC or PRV groups relative to non-IRAP treatment-matched individuals (Fig. 3a). Cumulatively, these data indicate that although a severe physiological disturbance has the potential to significantly increase transcription of inflammatory cytokines and thereby potentially inflammatory disease, the stresses associated with IRAP did not result in conditions that directly enhanced the capacity of either rhabdovirus (IHNV) or reovirus (PRV) to replicate or incur inflammatory disease in situations where inflammatory signaling was not already activated.

## Discussion

### Energetic implications of viral resistance vs tolerance

Transcriptomic analyses have identified strikingly divergent response profiles in sockeye salmon exposed to PRV versus IHNV, as well as between IHNV-resistant versus IHNV-susceptible individuals [22, 27]. Our study aimed to expand on these findings to determine if and to what extent such transcriptomic changes correlate with changes to steady-state energetics and aerobic performance.

For PRV, transcriptome analyses have indicated non-responsiveness during early infection and dissemination with only modest induction of viral recognition pathways during peak high-load (e.g., >10^9^ copies/mL) infections [22]. ISG transcriptional profiles in this study were congruent with previous observations and support the idea that PRV has a low virulence in sockeye salmon via its general ability to avoid host recognition despite extensive systemic replication. Correspondingly, it was perhaps unsurprising that we found aerobic performance and steady-state energetic requirements of PRV-infected fish to be similar to controls during the primary PRV replication and dissemination phases given that direct energetic parasitism by the virus was expected to be low [18].

Sockeye salmon were not, however, completely tolerant to extreme PRV loads [22], as evidenced by moderate ISG induction during high-load systemic PRV infection that was accompanied by a temporary and modest reduction in Hct, Hb concentration, and prolonged EPOC_dur_. The mechanistic basis for these temporary changes is unclear and they may be related. One possibility, for example, is that host-directed killing of PRV-infected red blood cells (i.e., a consequence of defensive countering by the host) temporarily decreased Hct and Hb, because mature PRV-infected red blood cells being removed contain slightly more Hb than the immature ones replacing them. A prolonged EPOC_dur_ could be a result of increased red blood cell fragility (i.e., a consequence of direct harm by the virus), which is another possible mechanism for mature red blood cell removal [31]. Regardless, the effects on EPOC_dur_ were small and temporary, a rather short window for a putative life-history tradeoff to occur, and would likely require maximal exercise to fully manifest a measurable aerobic cost [36]. This indicates, therefore, that host tolerance to non-pathogenic or low-virulent viral infection can be a highly efficient energetic strategy with limited life-history-associated aerobic performance risk. It also suggests that a combination of host tolerance and low viral virulence has the potential to be a commensal rather than parasitic relationship—an important paradigm to consider in the context of future non-disease-associated (orphan) virus discoveries.

For IHNV, transcriptome analyses have indicated that sockeye salmon experience global transcriptomic changes following exposure and that resistant and susceptible individuals have discrete profiles [27]. Primary resistance further appears to involve responses that encourage reduced cellular signaling rather than promoting classical innate antiviral responses or energetic expenditure [27]. Here, we identified a lower SMR in IHNV-resistant compared to IHNV-susceptible individuals, which indicates global transcriptomic changes do indeed manifest as a measurable reduction of steady-state energetic requirements. Notably, the reduced SMR associated with IHNV resistance, however, did not come at a further measurable cost of compromised aerobic capacities or performance. This would indicate that life-history tradeoffs associated with primary rhabdovirus resistance in salmon are unlikely to manifest directly as either energetic or aerobic shortcomings.

The high energetic efficiency of IHNV resistance demonstrated by sockeye in the face of potentially lethal infection in this study is not to say that this resistance is not without an indirect putative life-history cost. Increased resistance to one type of parasite can often lead to higher susceptibility to another [33, 37–39]. Environmental conditions can also dictate whether manipulating energetic requirements and a lower SMR could be both beneficial or harmful to long-term growth and survival [40–42]. Furthermore, it is unclear if inherent IHNV resistance, which for sockeye appears at least in part to be a heritable genetic factor [43, 44], is associated with random mutation, adaptational capacity, and/or dependent on environmental conditions. Continued explorations will be necessary to comprehensively (or at least foundationally) understand the energetics associated with parasite resistance, for which our methods present a practical paradigm. Nevertheless, transcriptome studies in multiple fish species in concert with our current findings suggest that reducing metabolic signaling, and by implication SMR, is a common viral resistance strategy in vertebrates [45, 46] and is also in line with integrated human-virus metabolic stoichiometric modeling predictions for a host rather than viral-optimal state [47].

For susceptible individuals unable to resist robust IHNV dissemination, our findings indicate that secondary resistance strongly integrates the interferon system of sockeye salmon, consistent with a previous study [34]. We further identified that increased IHNV susceptibility coincided with increased O_2crit_ and thus reduced tolerance to environmental hypoxia. Compromised O_2crit_, however, does not appear directly associated with activation of the interferon system (see discussion below), but rather may be a result of cellular necrosis caused by IHNV. Specifically, we interpret the decrease in Hct and increased prevalence of interstitial hematopoietic cell hyperplasia in this study to suggest mild increases in hemorrhaging necessitating hematopoiesis, a hallmark of IHNV-associated disease [48], which in turn is likely responsible for increased O_2crit_ via a statistically nonsignificant reduced mean corpuscular hemoglobin concentration of younger erythrocytes. This implies that although salmon are more acutely sensitive to low-oxygen conditions during a robust IHNV infection, this consequence is likely attributed to viral-induced tissue damage rather than interferon activation and thus a pathogen-specific phenomenon.

### Impact of interferon responses on homeostasis and respiratory function

The interferon system is a primary means by which vertebrates recognize viral infections and initiate antiviral defenses [49]. Disruption of the interferon signaling cascade typically increases host susceptibility to viral infection [50], whereas nonspecific activation of the interferon system can transiently enhance host resistance to subsequent viral insult [51]. The fact that robust interferon expression is not constitutively maintained in vertebrate systems clearly indicates that continuous deployment comes at an adverse cost to the host, likely either by increasing energetic requirement or as an increased risk for viral counter adaptation.

Our comprehensive investigations found no evidence to support that acute activation of the interferon antiviral response system in sockeye salmon compromises homeostasis or respiratory performance of the organism. This finding compliments data generated in a parallel respiratory study of Atlantic salmon (*Salmo salar*) following reoviral-induced interferon activation [28]. We interpret our results as indicating that the ancestral vertebrate autocrine and paracrine “antiviral state” created in localized tissues and cells as a result of interferon activation appears to be within the short-term allostatic capacity of the organism to maintain homeostasis and thus does not imply a life-history tradeoff in terms of aerobic or anaerobic performance. This is an important consideration in the current age of viral discovery and host transcriptional monitoring, given that viral recognition by the host has repeatedly been interpreted as an implicit sign of disease and/or harm [52–54]. Our work indicates that if a life-history tradeoff is not inherently implied via activation of the interferon system, then components of this system cannot be inferred to imply a viral-associated disease state or functional harm to the organism.

This does not necessarily contradict previous evidence that the interferon system can alter the capacity of cellular energetics [14] as the lack of aerobic changes in salmon in the present study indicates a broader allostatic capacity of the organism to maintain homeostasis rather than manipulation of a single cell’s energy expenditure. Our findings also do not negate that derived immunological responses integrated with the interferon system, such as the fever response in homeothermic mammals, may be energetically expensive as previously indicated [5, 55].

### Relationship of acute stress and viral resistance

Glucocorticoids are a major component of the vertebrate stress response and have diverse, cell-type-dependent actions on the immune system. Little is known about the interaction between these hormones and cytokines as far as how these interactions manifest changes in pathogen resistance or aerobic performance at the organism level. In this study, we observe that for both the sockeye-PRV and sockeye-IHNV challenge models, acute IRAP stress (an activity expected to generate substantial if not maximal plasma glucocorticoid levels [29, 35]) 4 days after virus infection did not produce a measurable effect on peak ISG transcription or viral load. This indicates that for sockeye experiencing severe short-term fright responses during a life-threatening viral infection, the associated modifications to immune function (e.g., decreased interferon transcription) do not appear to exceed the allostatic capacity of the organism to maintain effective viral resistance (e.g., increased ISG transcription) or to notably affect the outcome of viral replication. Thus, not only are sockeye smolts able to exert maximum flight capabilities while fighting an IHNV infection, an exhaustive flight response does not appear to incur an immunological cost with regard to resisting or ultimately clearing IHNV. In fact, our data suggests that IRAP stress may even have been somewhat advantageous in reducing IHNV loads.

Although stress often also appears positively associated with inflammatory disease states [56, 57], stress hormones are widely known to be strong anti-inflammatory compounds by suppressing pro-inflammatory expression of cytokines in immune cells [58]. This identifies a degree of complexity for invoking a stress response beyond the immediate circulation of plasma glucocorticoids such as cortisol. For PRV, the fact that acute IRAP stress did not affect tolerance in sockeye indicates that the host-virus interaction—and specifically inflammatory signaling—was unaffected by an acute stress event during infection. For IHNV, on the other hand, we saw a significant increase in inflammatory cytokine transcription subsequent to an IRAP stress event. These data together imply that in the sockeye salmon model, stress has the capacity to enhance underlying inflammatory signaling such as when initiated by IHNV, but is not sufficient to induce an inflammatory response de novo, such as during a PRV infection or in otherwise healthy individuals. We speculate that this effect may, at least in part, be caused by the reduced transcription of interferon in sockeye, which often acts in restraining inflammation [13]. This as well as alternative mechanisms may prove exciting avenues for further exploration toward defining the potential for enhancement (but perhaps not de novo activation) of inflammation by acute stress in other vertebrate systems.

### Remaining knowledge gaps and limitations

The findings of this study provide an important demonstration that viral resistance, including the deployment of an interferon signaling cascade, can be energetically inexpensive in a vertebrate system without compromising physical organism performance or imposing an overt energetic demand. However, it must also be acknowledged that the findings of this study should not necessarily be applied as representative of parasite resistance in vertebrate systems at large. Indeed, the complex systems vertebrates employ for tailoring cytokine responses identifies that there will almost certainly be variations across vertebrate-pathogen interactions—the implementation of energetically costly fever responses of mammals being a good example. For poikilothermic vertebrates such as fish, reptiles, and amphibians, environmental temperature significantly affects the timing of immune responses and thus may also incur differential energetic costs in relation to parasite resistance in many situations. In this study, we conducted our model experiments in a conducive temperature range for PRV, IHNV, and salmon immune functioning [22, 27, 31, 59]. However, temperature manipulations can often give advantage to either the host or the pathogen and it will be interesting for future studies to consider the energetic cost of resistance in this context—particularly the role that behavior fever may play in modulating the energetic tradeoffs associated with parasite resistance.

## Conclusion

In this study, we tested three hypotheses relating to the energetic costs of viral resistance and tolerance in this vertebrate system. We first hypothesized that mounting resistance incurs a metabolic cost or limitation. Contrary to this hypothesis, we found that effective resistance against a life-threatening rhabdovirus infection and tolerance to a high-load reovirus infection both were highly energetically efficient strategies that incurred minimal metabolic costs and potential life-history-associated energetic tradeoffs. Second, we hypothesized that induction of the innate antiviral interferon system compromises homeostasis. This hypothesis was also rejected, as we found no evidence to support that robust systemic activation of the interferon system engenders an energetic requirement sufficient to surpass the allostatic capacity of fish to compromise homeostasis or respiratory performance. Lastly, we hypothesized that antiviral defenses are weakened by acute stress. This hypothesis too was rejected by data indicating that acute stress experienced during respiratory assessments did not weaken host antiviral defenses or promote viral replication. However, data regarding inflammatory stimulation in response to IHNV infection did suggest a potential for disease intensification due to acute stress contingent on the presence of preexisting inflammatory responses within the host. More importantly, we demonstrated that, as least in some cases, reduced cellular signaling but not innate antiviral responses promote viral resistance. Together, these findings demonstrate that fundamental innate vertebrate defense strategies, even in the face of potentially life-threatening viral exposures, have high energetic efficiency and provide a foundational paradigm for further assessing life-history-associated costs of parasite resistance in a vertebrate system.

## Methods

### Animal husbandry

A cohort of Pitt River sockeye salmon (approximately 1 g body mass as determined by bulk weight estimates) was obtained from Inch Creek Salmonid Enhancement Program hatchery in Dewdney, British Columbia. Fish were transported to the Fisheries and Oceans Canada Pacific Biological Station (49.2106° N, 123.9554° W) where all experiments were conducted. A pre-transport screening of 20 fish did not detect PRV by quantitative PCR (qPCR), nor was any other virus propagated on CHSE-214 or EPC diagnostic fish cell lines. Fish were reared in 6–8°C municipal de-chlorinated, UV-irradiated freshwater for 12 months on EWOS age-appropriate dry pellet rations (1.0% body weight per day) after which they were transitioned to sand-filtered, UV-irradiated, 10°C seawater (salinity 28–32 ppt) over a 2-week period. Four hundred fish (∼110 g body mass per fish by bulk weight estimate) were then divided equally into eight 350-L circular experimental tanks (50 fish per tank) supplied with 15 L per min flow-through, sand-filtered, UV-irradiated 10–11°C seawater and held for 45–55 days prior to viral challenge. Fish were fed EWOS dry pellet rations (0.5% body weight per day) throughout the study and dissolved oxygen was maintained above 90% air saturation (>8.2 mg per L) in the culture system.

### Virus propagation and purification

#### PRV

PRV has resisted all attempts of in vitro propagation [31]. Consequently, purified PRV infectious particles were obtained by collecting PRV-infected blood from a commercially farmed Atlantic salmon (isolation 16-005ND [23];). Blood was sonicated, clarified via 2000*g* centrifugation, and i.p. injected into a cohort of 10 naïve Atlantic salmon as previously described [23]. Following a 3-week holding period at 11°C in seawater, fish were euthanized and peripheral blood collected and sonicated, and viral particles purified via cesium chloride gradient ultracentrifugation [60]. Following 24-h dialysis in modified Dulbecco’s phosphate-buffered saline *sans* magnesium and calcium, particle purity was visualized via electron microscopy and reverse-transcribed copies of PRV L1 genomic material were enumerated using qPCR as a proxy of viral titer [23]. Glycerol was added to a final volume of 10% and aliquots were stored at −80°C.

#### IHNV

The BC93-057 isolate of IHNV (genogroup U) was propagated and quantified using *Epithelioma papulosum cyprini* (EPC) cells using standard techniques [26]. Specifically, virus stock with less than three in vitro passages was used to inoculate a monolayer of EPC cells at a multiplicity of infection of 0.001. Cells were cultured in Minimal Essential Media supplemented with 10% fetal bovine serum (MEM-10) at 15°C. IHNV was harvested by centrifugation of the culture media at 3000*g* for 15 min as soon as the total cytopathic effect was observed (7 days post-infection). Viral titer within the supernatant was determined by plaque assay using EPC monolayers [61] before being stored at −80°C.

### Experimental infection

PRV and IHNV stocks were thawed and media were added to achieve a final concentration of 1% glycerol, 9% Dulbecco’s phosphate-buffered saline (*sans* magnesium and calcium), and 90% MEM-10. These stocks were then diluted 1:100–1:10,000 with Hanks balanced salt solution to achieve a targeted dose of either 1 × 10^6^ PRV reverse-transcribed L1 genomic copies or 5 × 10^5^ pfu of IHNV per 100 μL. Media without virus was identically prepared to act as a vehicular saline control (SC). Duplicate tanks of sockeye salmon (50 fish per tank) were anesthetized in 75 mg per L tricaine mesylate (MS-222) and each fish administered a 100 μL intraperitoneal injection of either the PRV, IHNV, or SC inoculate. Treatments were administered by staggered 5-day increments so as to complete IRAP assessments, which requires 4 days per treatment group. Days post-challenge (dpc) were therefore compared relative to each other rather than by calendar day (see Additional file 2).

Because a dichotomy of resistance and susceptibility was expected for IHNV exposure [27], the sample size following the first IRAP assessments was increased by means of challenging a second cohort of sockeye salmon (duplicate tanks of 50 fish per tank) with IHNV 5 days after the first IHNV-infected cohort. Thus, at 1 wpc, IHNV-challenged samples were collected from 16 individuals post-IRAP and 16 individuals directly from the tank (non-IRAP) whereas all other sampling events consisted of samples being collected from eight individuals post-IRAP and eight individuals from the holding tank for each respective treatment (see Additional file 2).

### Respiratory performance

IRAP assessments lasted a total of 4 days and were conducted at 1, 4, and 9 wpc (4–8, 25–29, and 60–64 dpc, respectively). The IRAP protocol used an 8-chamber intermittent-flow respirometry system as previously described [15, 28–30, 62]. Specific to the present study, the 8-chamber system was immersed in a 600 L flow-through seawater bath to maintain a constant temperature (11 ± 0.5 °C). Fish, which had been fasted for 72 h, were individually hand chased in 22-L aquaria to exhaustion via a standardized 6-min stimulation followed by a 1 min air exposure before being individually placed into a respirometer. ṀO_2_ measurements began immediately using cycling conditions consisting of a 35-s flush period, a 50-s sealed stabilization period, and a 110-s sealed measurement period. After 1 h, and as peak oxygen uptake (ṀO_2max_) subsided, water cycling conditions were modified to a 65-s flush period, a 110-s sealed stabilization period, and a 345-s sealed measurement. These conditions maintained >85% air saturation at all times in all chambers and were refreshed to >98% air saturation with each flush during the quiescent holding period.

Fish remained undisturbed in a normoxic state for 4 days, during which EPOC was completed and ṀO_2_ was recorded for quiescent fish every 8.7 min to ensure that SMR could be reliably estimated from > 600 individual ṀO_2_ data points per fish [63]. An IRAP assessment was concluded by inducing hypoxia (40% air saturation) in the respirometer over a 45-min period using nitrogen gas supplementation, followed by a further decrease of oxygen at a slower rate of 0.15% air saturation per min until each fish lost control of dorso-ventral equilibrium. During the hypoxia exposure, water cycling conditions were adjusted to a 50-s flush period, an 85-s sealed stabilization period, and a 225-s sealed measurement. Following loss of equilibrium, fish were euthanized for immediate blood and tissue sample collection. The system was flushed with 1% Virkon™ S disinfectant for 12–18 h followed by 11°C seawater for 6h between each IRAP assessment.

### Sample collection and processing

Blood (1 mL by caudal puncture) and tissue (by dissection) were collected from all fish immediately at the termination of IRAP assessments and from an equal number of time-matched fish sampled by dip net directly from the holding tanks (non-IRAP) immediately following euthanasia by a percussive blow to the head. Thus, tissue samples from 16 fish (8 IRAP; 8 non-IRAP) were collected at 8, 29, and 64 dpc for the three treatment groups. Also, an additional 16 IHNV-challenged fish (8 IRAP; 8 non-IRAP) were sampled at 8 dpc as indicated above (see Additional file 2). Similar to Zhang et al. [28], 100 μL blood was immediately frozen in liquid nitrogen for PRV screening and host gene expression analysis by qPCR. Hematocrit was determined from 10 μL blood transferred to a sodium-heparin treated Fisherbrand™ micro-hematocrit tube and spun at 15,000×*g* for 10 min. The remaining blood was transferred to a heparinized vacutainer held on ice (∼24 h) and used to determine oxygen binding, blood pH, and hemoglobin concentration as previously described [28]. A portion of the kidney was removed and frozen in liquid nitrogen for IHNV screening and host gene expression analysis. Hearts were bisected longitudinally and one half preserved in 10% neutral buffered formalin along with approximately 100–200 mg of the kidney. These preserved tissues were transferred to isopropanol following 24–48 h and processed for histopathological examination as previously described [64, 65]. Microscopic findings were assessed by pathologists blinded to treatment. Most findings were semi-quantitatively scored as none (0), mild/small amounts (1), moderate (2), or severe/abundant (3). One pathologist examined all of the samples; a second (reviewing) pathologist independently selected and analyzed sections from one of every 10 fish for which consensus scores are reported (Additional file 2).

### Viral screening

PRV nucleic acid was detected using previously described methods [23] where viral RNA was extracted from 100 μL homogenized blood in TRIzol Reagent (Life Technologies) following the manufacturer’s instructions. A portion of eluted RNA (1.0 μg) was denatured for 5 min at 95 °C and immediately cooled to 4 °C before being reverse-transcribed using a High-Capacity cDNA Reverse Transcription kit (Life Technologies). One microliter of resulting cDNA was used directly as a template for qPCR analysis in a StepOne-Plus real-time detection system (Applied Biosystems) with previously described primers and TaqMan probe [21]. Each 15-μL reaction contained 400 nM primers and 300 nM TaqMan probe, 1X TaqMan Universal Master Mix, and 1 μL cDNA template. Cycling conditions included an initial incubation of 95 °C for 10 min followed by 40 cycles of 95 °C for 10 s and 60 °C for 20 s. Samples were assayed in duplicate and were considered positive if both technical replicates reported a Ct value < 40 cycles. Absolute PRV quantification was determined by serial dilution of a 482-bp double-stranded DNA gBLOCK fragment (Integrated DNA Technologies) [21] run in duplicate on each qPCR plate.

IHNV nucleic acid was detected using methods similar to those described by Purcell et al. [66] and Polinski et al. [22] where RNA was extracted following manufacturer’s instructions using ~50 mg of homogenized kidney in TRIzol Reagent. A portion of eluted RNA (1.0 μg) was denatured for 1 min at 80 °C and immediately cooled to 4 °C before being reverse-transcribed using a High-Capacity cDNA Reverse Transcription kit. Resulting cDNA was used directly as a template for qPCR analysis in a StepOne-Plus real-time detection system using previously described primers and TaqMan probe [22]. Each 15-μL reaction contained 900 nM primers and 200 nM TaqMan probe, 1X TaqMan Universal Master Mix, and 1 μL cDNA template. Cycling conditions included an initial incubation of 50°C for 2 min followed by 95 °C for 10 min, and then 40 cycles of 95 °C for 15 s and 60 °C for 1 min. Samples were assayed in duplicate and were considered positive if both technical replicates reported a Ct value < 40 cycles. Absolute IHNV quantification was determined in each instance by serial dilution of a gBLOCK fragment as for PRV [22].

### Immune gene expression

Gene expression analysis was conducted similar to previously described methods [21, 28] where a portion (5 μg) of total RNA extracted from kidney tissues that was not reverse-transcribed for the detection of the virus was purified using 2 U of DNase I (Life Technologies) at 37°C for 45 min followed by RNeasy MinElute Cleanup (Qiagen) as per manufacturer’s instructions. Quality was visualized on a 1% bleach denaturing gel and 1 μg of each sample was reverse-transcribed using a High-Capacity cDNA Reverse Transcription Kit without RNase inhibitor in which the Random Primer mix was substituted with 50μM Olido d(T)_16_. All real-time qPCR analyses were conducted on a StepOne-Plus real-time detection system using SYBR green chemistry. Each reaction consisted of 1X SYBR master mix (Life Technologies), forward and reverse primers (500 nM each; see Additional file 2), and 1.5-μL cDNA template to a final volume of 15 μL. Samples were assayed in duplicate with a five-step, fourfold dilution series of pooled cDNA included in each run to calculate amplification efficiency, linearity, relative quantity and to provide inter-run calibration. Cycling conditions consisted of an initial activation of DNA polymerase at 95°C for 10 min, followed by 40 cycles of 5 s at 95°C, 25 s at 60°C, and 10 s at 72°C. A melt-curve analysis was conducted for each run to ensure amplification specificity. All gene expression data were normalized to β-actin transcription which has previously shown stable expression following both PRV and IHNV infection of sockeye salmon [22].

### Statistical analysis

Survival of sockeye salmon following either PRV or IHNV injection was compared to SC via the log-rank Mantel-Cox test. Comparison of histopathological scores in PRV- and IHNV-injected sockeye salmon was exploratorily assessed relative to an “ideal” standard of no pathology (usually score 0) using Kruskal-Wallis rank tests followed by Dunn’s multiple comparison tests for each of 71 putative pathological conditions noted in Additional file 2. In the 21 instances where the median score was potentially suggested to be non-ideal (multiplicity adjusted p-value <0.2), the pathology scores noted in the viral treatment groups were then compared to time-matched SC to identify treatment-specific relevance using the same statistical methods (Additional file 1: Table S1; a priori power analysis > 0.69 using G*Power 3.1.9.7 [67] at α = 0.05, f = 0.8).

IRAP, blood oxygen-carrying capacity, and body condition data were individually assessed by 2-way ANOVA (a priori power analysis > 0.85 at α = 0.05, f = 0.4) followed by Dunnett multiple comparisons tests for both PRV- and IHNV-injected treatment groups relative to SC in a time-point-specific manner (Additional file 1: Fig S1, S2 & S4). P-values were adjusted for familywise error but no false discovery rate calculations were applied over the suite of comparisons. T_0.5ṀO2max_, T_0.8ṀO2max_, and AOD were log-transformed prior to analysis and 0.001 was added to all individual T_0.5ṀO2max_ and T_0.8ṀO2max_ measures to eliminate undefined values. IRAP and blood oxygen-carrying capacity measures were further compared to log-viral RNA quantities via Spearman correlations [a priori power analysis > 0.81 at α = 0.05, ρ(H_a_) = 0.5, ρ(H_o_) = 0].

Gene expression data were normalized to β-actin transcription and the normalized quantities were corrected to the minimum value (CNRQ). CNRQ values of IHNV- and PRV-injected fish were then compared to time-matched SC following log-transformation by two-way ANOVA (a priori power analysis > 0.85 at α = 0.05, f = 0.4) followed by Dunnett multiple comparison tests. Gene expression data were also compared to log-viral RNA quantities via Spearman correlations [a priori power analysis > 0.81 at α = 0.05, ρ(H_a_) = 0.5, ρ(H_o_) = 0]. For all correlative analysis, biological relevance was considered likely only for trends which extended to more than one standard deviation of the mean SC value.

## Supplementary Information

**Additional file 1. **Polinski et al. 2021 Supplemental Tables and Figures. Adobe PDF (.pdf) file containing the following supplemental tables and figures referenced in the text: **Table S1.** – Statistical analysis relating to histopathological condition scores; **Fig. S1**. – Violin plots of respiratory indices and body condition measured at each sample timepoint; **Fig. S2**. – Violin plots of the corrected normalized relative quantity (CRNQ) for five mRNA gene transcripts measured at each sample timepoint; **Fig. S3**. – Correlation of il1b CNRQ relative to either PRV blood or IHNV kidney log transcriptional load; **Fig. S4**. – Violin plots of five blood oxygen associated parameters measured at each sample timepoint; **Fig. S5**. – Correlation of log IHNV RNA kidney transcription relative to standard metabolic rate and critical oxygen saturation at 1 wpc peak infection; **Fig. S6**. – IHNV and PRV pilot challenge data.

**Additional file 2.** Polinski et al. 2021 Collected and generated data. Microsoft Excel (.xlsx) file containing all collected and generated data pertaining to this study: **Tab 1** – Sample inventory; **Tab 2** – Timeline and mortality log; **Tab 3** – Histopathology scoring; **Tab 4** – qPCR primers.

## Data Availability

All data supporting the conclusions of this article are included within Additional file 2.
