# Author Correction: Innate antiviral defense demonstrates high energetic efficiency in a bony fish

**DOI:** 10.1186/s12915-023-01594-2

**Published:** 2023-05-19

**Authors:** Mark P. Polinski, Yangfan Zhang, Phillip R. Morrison, Gary D. Marty, Colin J. Brauner, Anthony P. Farrell, Kyle A. Garver

**Affiliations:** 1 Fisheries and Oceans Canada Pacific Biological Station, 3190 Hammond Bay Road, Nanaimo, V9T6N7 Canada; 2 Faculty of Land and Food Systems, University of British Columbia, MCML 344-2357 Main Mall, Vancouver, V6T1Z4 Canada; 3 Department of Zoology, University of British Columbia, 6270 University Blvd, Vancouver, V6T1Z4 Canada; 4 Animal Health Centre, Ministry of Agriculture, Food and Fisheries, 1767 Angus Campbell Rd, Abbotsford, V3G2M3 Canada

**Correction: BMC Biol 19, 138 (2021)**

**https://doi.org/10.1186/s12915-021-01069-2**

The original article has been updated with the following corrections.

**Correction 1:**

The original article [1] contained an accidental omission of two data points in the 1 Week post challenge (Wpc) saline control column of the excess post-exercise oxygen consumption (EPOC) panel in Fig S1 (Fish ID # 14 & 15, Additional file 2).

The EPOC panel as originally published in Fig. S1:
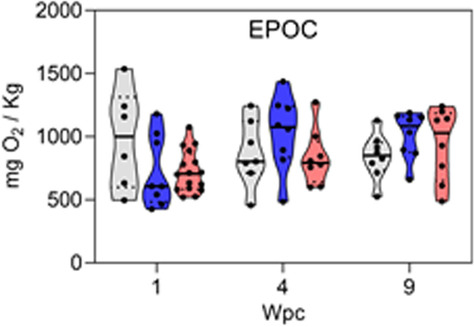


The corrected EPOC panel in Fig. S1:
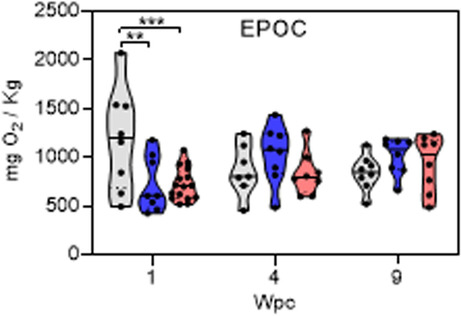


Although this error does not affect the conclusions in the original article, it does alter statistical probability estimates in Fig S1 for comparing EPOC between treatment groups at 1 Wpc which warrants a correction to Fig. S1 and the following textual correction in the article:

In the results section **“Sockeye salmon tolerated high-load PRV infections with only mild transient consequences to oxygen transport and exhaustive chase recovery”**,

*“Respiratory performance of sockeye salmon was largely uncompromised as a result of PRV infection. Notably, 14 of the 15 respiratory indices were unchanged relative to time-matched control fish. However, one minor temporary respiratory change occurred. The duration of excess post-exercise oxygen consumption (EPOCdur) following an exhaustive chase was prolonged by 43% (95% CI* = *0.1–85%; p* = *0.05) only at the early viral persistence phase (9 wpc; see Additional file 1: Fig. S1) without any significant effect on ṀO2max or total EPOC.”*

Now reads,

*“Respiratory performance of sockeye salmon was largely uncompromised as a result of PRV infection. Notably, 13 of the 15 respiratory indices were unchanged relative to time-matched control fish. However, two putative respiratory changes were observed. High variability and elevated (mean 64%; 95% CI* = *21-107%; p* < *0.002) EPOC was noted within SC fish at 1 WPC relative to both PRV and IHN. The duration of excess post-exercise oxygen consumption (EPOCdur) following an exhaustive chase was also prolonged by 43% (95% CI* = *0.1–85%; p* = *0.05) at the early PRV persistence phase (9 wpc; see Additional file 1: Fig. S1) without any significant effect on ṀO2max or total EPOC.”*

**Correction 2:**

The original article stated that PRV prevalence in challenged fish was 100% at 4 & 9 Wpc. Although this statement is true, data presented in Additional file 2 indicated PRV was not detected in blood samples from fish 84 or fish 143 by qPCR at 4 & 9 Wpc, respectively. This apparent contradiction warrants further explanation. Suspecting these results to be in error based on ISG expression presented in Additional file 2 and epidemiological precedent from past published challenge trials, kidney samples from these two fish were screened for PRV by qPCR and were robustly infected with PRV (> 10^5^ copies/µg total RNA). This verified our suspicion of a false-negative blood screening results for these two fish. The following explanatory comments have been added to the ID cell of fish 84 and 143 in Additional file 2 and to the main text to eliminate any potential confusion relating to the reported PRV-negative blood results:

In Additional file 2, cell 84,

*“Although PRV was not detected in this peripheral blood sample by qPCR, PRV was readily detected (5.4 x 10^6 copies/µg total RNA) in a kidney sample from the same fish using the same assay. We consider the blood result to be in error and that this fish was infected with PRV.”*

In Additional file 2, cell 143,

*“Although PRV was not detected in this peripheral blood sample by qPCR, PRV was readily detected (1.9 x 10^5 copies/µg total RNA) in a kidney sample from the same fish using the same assay. We consider the blood result to be in error and that this fish was infected with PRV.”*

In the results section **“Sockeye salmon tolerated high-load PRV infections with only mild transient consequences to oxygen transport and exhaustive chase recovery”,**

*“At 1 week post-challenge (wpc), 6 of 16 fish (38%) had developed a moderate systemic blood infection and by 4 wpc all sampled fish were systemically infected with PRV with a mean *(± *SE) blood load of 4.9 (*± *1.5)* × *10*^*7*^* reverse-transcribed L1 genome segment copies per μg total blood RNA—equivalent to approximately 1.2* ± *0.4* × *10*^*10*^* copies per mL blood. At 9 wpc, PRV infection prevalence remained at 100% and mean infection intensity remained high (1.6* ± *0.4* × *10*^*7*^* copies per μg blood RNA or approximately 3.5* ± *1.0* × *10*^*9*^ *copies per mL).*”

Now reads,

“*At 1 week post-challenge (wpc), 6 of 16 fish (38%) had developed a moderate systemic blood infection. At 4 wpc PRV infection was detected in the blood of 15 of 16 sampled fish with a mean (*± *SE) blood load of 4.9 (*± *1.5)* × *10*^*7*^* reverse-transcribed L1 genome segment copies per μg total blood RNA—equivalent to approximately 1.2* ± *0.4* × *10*^*10*^* copies per mL blood. One fish had a negative blood result but kidney tested positive at 5.4 x 10*^*6*^* copies per μg total kidney RNA thus we consider this fish to have been systemically infected. At 9 wpc, PRV infection prevalence remained at 100% and mean infection intensity remained high (1.6* ± *0.4* × *10*^*7*^* copies per μg blood RNA or approximately 3.5* ± *1.0* × *10*^*9*^* copies per mL) in 15 of 16 fish, and 1.9 x 10*^*5*^* copies per μg total kidney RNA was detected in the 16th fish.*”

**Correction 3:**

In the methods section, we would like to clarify that power calculations were specific to the main effect analyses and not to subsequent post hoc multiple comparison testing.

In the methods section **“Statistical Analysis”**,

“*IRAP, blood oxygen-carrying capacity, and body condition data were individually assessed by 2-way ANOVA followed by Dunnett multiple comparisons tests for both PRV- and IHNV-injected treatment groups relative to SC in a time-point-specific manner (Additional file 1: Fig S1, S2 & S4; a priori power analysis* > *0.85 at α* = *0.05, f* = *0.4).”*

Now reads,

*“IRAP, blood oxygen-carrying capacity, and body condition data were individually assessed by 2-way ANOVA (a priori power analysis* > *0.85 at α* = *0.05, f* = *0.4) followed by Dunnett multiple comparisons tests for both PRV- and IHNV-injected treatment groups relative to SC in a time-point-specific manner (Additional file 1: Fig S1, S2 & S4).”*

And,

*“CNRQ values of IHNV- and PRV-injected fish were then compared to time-matched SC following log-transformation by two-way ANOVA followed by Dunnett multiple comparison tests (a priori power analysis* > *0.85 at α* = *0.05, f* = *0.4).”*

Now reads,

*“CNRQ values of IHNV- and PRV-injected fish were then compared to time-matched SC following log-transformation by two-way ANOVA (a priori power analysis* > *0.85 at α* = *0.05, f* = *0.4) followed by Dunnett multiple comparison tests.”*

**Correction 4:**

Although stated elsewhere in the article, information on funder involvement was absent from the declaration of competing interests in the original article, and we should like to restate this information in a corrected declaration along with some additional explanation to address potential perceived bias.

The **“Competing interests”** declaration, stating *“The authors declare that they have no competing interests.”*

Now reads,

*“The authors declare that they have no competing interests. The British Columbia Salmon Farmers Association provided in kind (experimental organisms) as well as monetary (operations and maintenance) contributions to Fisheries and Oceans Canada for this project and did not participate in the experimental study design, data collection and analysis, preparation of the manuscript, decision to publish, hold no intellectual property rights associated with data or procedures developed in this study, and did not provide financial or nonfinancial support to individual authors.”*

## Supplementary Information

**Additional file 1.**

**Additional file 2.**
