# Supplementary material for: Innate antiviral defense demonstrates high energetic efficiency in a bony fish
Source: BMC Biol. 2021 Jul 13;19:138. doi: 10.1186/s12915-021-01069-2 (PMC8276435; doi:10.1186/s12915-021-01069-2)
Supplement: Supplementary file 1 — Additional file 1. Polinski et al. 2021 Supplemental Tables and Figures. Adobe PDF (.pdf) file containing the following supplemental tables and figures referenced in the text: Table S1. – Statistical analysis relating to histopathological condition scores; Fig. S1. – Violin plots of respiratory indices and body condition measured at each sample timepoint; Fig. S2. – Violin plots of the corrected normalized relative quantity (CRNQ) for five mRNA gene transcripts measured at each sample timepoint; Fig. S3. – Correlation of il1b CNRQ relative to either PRV blood or IHNV kidney log transcriptional load; Fig. S4. – Violin plots of five blood oxygen associated parameters measured at each sample timepoint; Fig. S5. – Correlation of log IHNV RNA kidney transcription relative to standard metabolic rate and critical oxygen saturation at 1 wpc peak infection; Fig. S6. – IHNV and PRV pilot challenge data. [file 12915_2021_1069_MOESM1_ESM.docx]

Supplemental Tables and Figures

Innate antiviral defense demonstrates high energetic efficiency in a bony fish

Mark P. Polinski^1†*^, Yangfan Zhang^2†^, Phillip R. Morrison^3^, Gary D. Marty^4^, Colin J. Brauner^3^, Anthony P. Farrell^2,3^, Kyle A. Garver^1*^

^1^ Aquatic Diagnostics and Genomics Division, Pacific Biological Station, Fisheries and Oceans Canada, Nanaimo, BC, Canada, ^2^ Faculty of Land and Food Systems, The University of British Columbia, Vancouver, BC, Canada, ^3^ Department of Zoology, The University of British Columbia, Vancouver, BC, Canada, Animal Health Centre, Ministry of Agriculture, Food and Fisheries, 1767 Angus Campbell Rd, Abbotsford, BC, Canada

*** Correspondence:** Mark P. Polinski: [Mark.Polinski@dfo-mpo.gc.ca](mailto:Mark.Polinski@dfo-mpo.gc.ca)

Kyle A. Garver: [Kyle.Garver@dfo-mpo.gc.ca](mailto:Kyle.Garver@dfo-mpo.gc.ca)

^†^ Equal contributors

**Article citation:**

Polinski MP, Zhang Y, Morrison PR, Marty GD, Brauner CJ, Farrell AP and Garver KA (2021) Innate antiviral defense demonstrates high energetic efficiency in a bony fish. *BMC Biology* 19, 138*.*

**Table S1**. Histopathological condition scores (see Polinski et al. 2021 Additional file 2 – recorded and generated data) were assessed in either PRV or IHNV injected fish relative to an “ideal” standard of no lesions (typically score of 0) by Kruskal-Wallis rank tests followed by Dunn’s multiple comparison tests for each of 71 putative pathological conditions. Conditions where the mean was putatively less than ideal (adjusted p-value < 0.2) were then compared to time-matched SC using the same statistical methods to indicate potential treatment-specific effect.

^a^ **Kidney abbreviations**: ISH = interstitial (hematopoietic) cell hyperplasia; TEP = tubular epithelial protein (intracytoplasmic); HEM = interstitial hemorrhage/congestion. **Heart abbreviations**: ENL = endocarditis/myocarditis, lymphohistiocytic. **Liver abbreviations**: CPL = cholangitis/pericholangial leukocytes; FPL = focal/multifocal parenchymal leukocytes; PVL = perivascular leukocytes

**Fig. S1**. Violin plots (line = median; dotted lines = 25^th^ & 75^th^ quartiles) of respiratory indices and body condition factor (k) measured at 3 discrete sampling events. Significant difference (* p < 0.05; ** p < 0.01; *** p < 0.001) in mean value between saline injected (SC; grey) and either PRV injected (PRV; blue) or IHNV injected (IHNV; red) treatment groups are indicated at each time point as determined by 2-way ANOVA and Dunnett’s multiple comparison tests. A log transformation of 0.5_ṀO2max_, T0.8_ṀO2max_ and AOD measures was applied prior to statistical comparison. Body conditions was assessed in fish sampled directly from the experimental holding tank (non-IRAP) or following 5 days of respiratory assessments (IRAP). ṀO_2max_ = maximum metabolic rate; SMR = standard metabolic rate; AAS = absolute aerobic scope; FAS = factorial aerobic scope; EPOC = excess post-exercise oxygen consumption; EPOC_dur_ = EPOC duration; RMR = routine metabolic rate; T0.5_ṀO2max_ = time spent above 50% of ṀO_2max_; T0.8_ṀO2max_ = time spent above 80% ṀO_2max_; O_2crit_ = critical oxygen level, ILOS = incipient lethal oxygen saturation; SOD = scope of oxygen deficit; FSOD = factorial SOD; AOD = accumulated oxygen deficit.


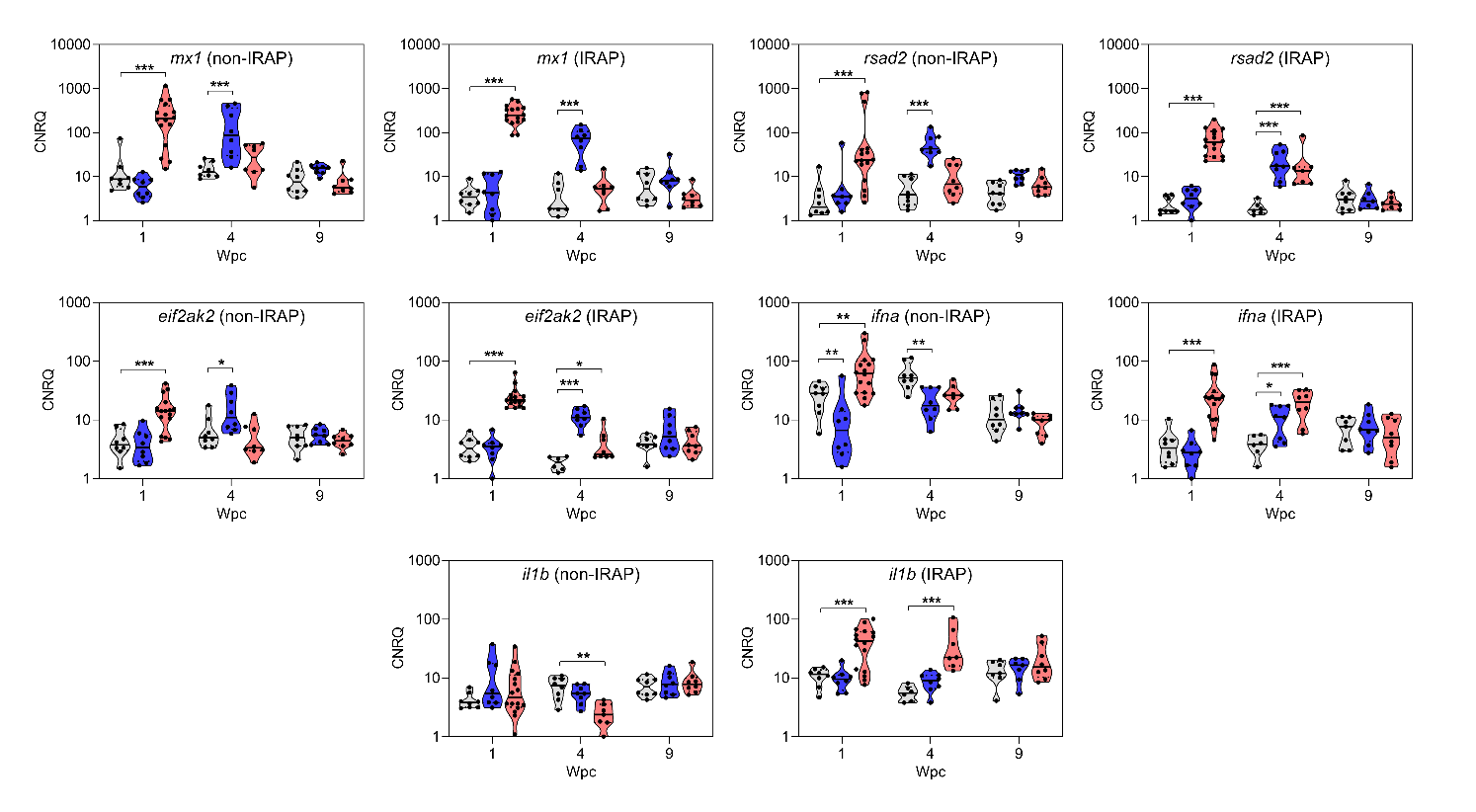


**Fig. S2**. Violin plots (line = median; dotted lines = 25th & 75th quartiles) of the corrected normalized relative quantity (CRNQ) for five mRNA gene transcripts measured at 3 discrete sampling events. * p < 0.05; ** p < 0.01; *** p < 0.001 differences in mean CNRQ between saline injected (SC; grey) and either PRV injected (PRV; blue) or IHNV injected (IHNV; red) treatment groups are indicated at each time point as determined by 2-way ANOVA and Dunnett’s multiple comparison tests following log transformation. *mx1* = mRNA transcript of Myxovirus Resistence-1 protein; *rsad2* = mRNA transcript of Viperin protein; *eif2ak2* = mRNA transcript of Protein Kinase-R protein; *ifna* = mRNA transcript for type-1 Interferon isoform-a protein; *il1b* = mRNA transcript of Interlukin-1β protein.


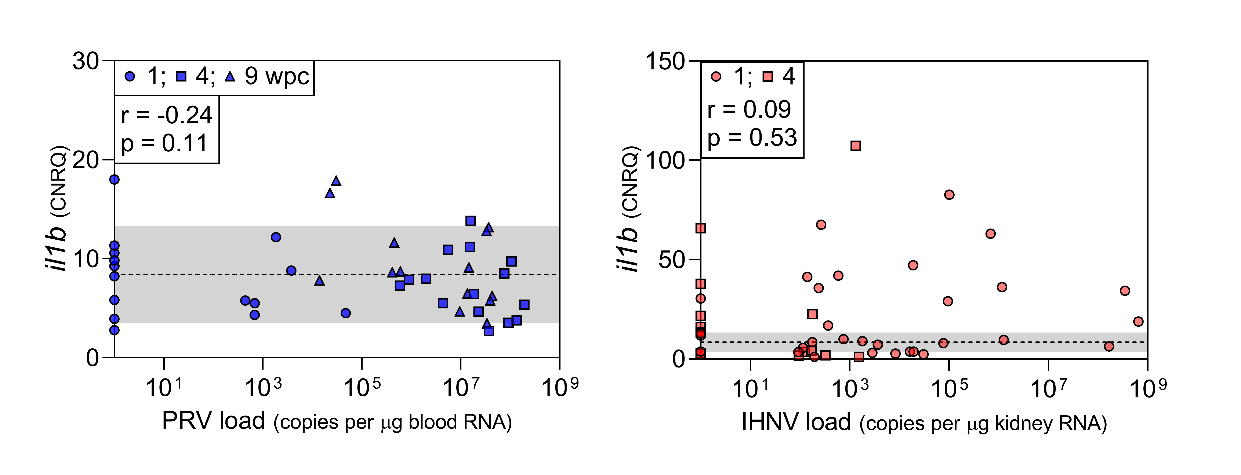


**Fig. S3.** Correlation of *il1b* CNRQ relative to either PRV blood or IHNV kidney log transcriptional load presented with Spearman R and associated p value statistics. The mean (dotted line) ± SD (shaded) of *il1b* expression in SC fish is provided for reference. *il1b* = mRNA transcripts of Interlukin-1β protein.


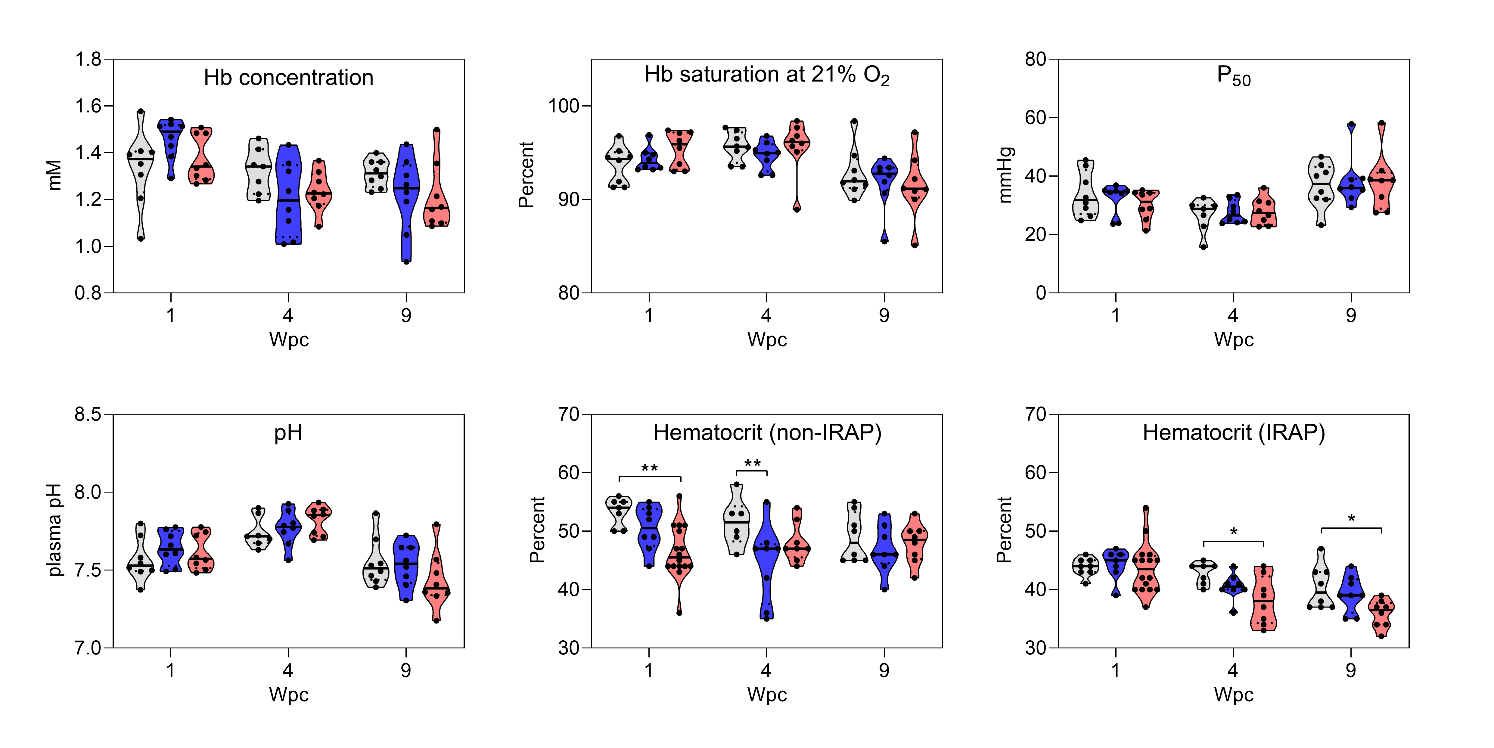


**Fig. S4**. Violin plots (line = median; dotted lines = 25th & 75th quartiles) of five blood oxygen associated parameters measured at 3 discrete sampling events. Significant variation (* p < 0.05; ** p < 0.01; *** p < 0.001) in mean value between saline injected (SC; grey) and either PRV injected (PRV; blue) or IHNV injected (IHNV; red) treatment groups are indicated at each time point as determined by 2-way ANOVA and Dunnett’s multiple comparison tests.

**Fig. S5**. Correlation of log IHNV RNA kidney transcription relative to standard metabolic rate (SMR) and critical oxygen saturation (O_2crit_) at 1 wpc peak infection. Thresholds of 10^3^ IHNV copies per ug, 64 mg 0_2_/h/Kg SMR, and 16% O_2crit_ (dotted lines) are used to define IHNV resistant (lower left quadrant) vs susceptible (upper right quadrant) individuals.


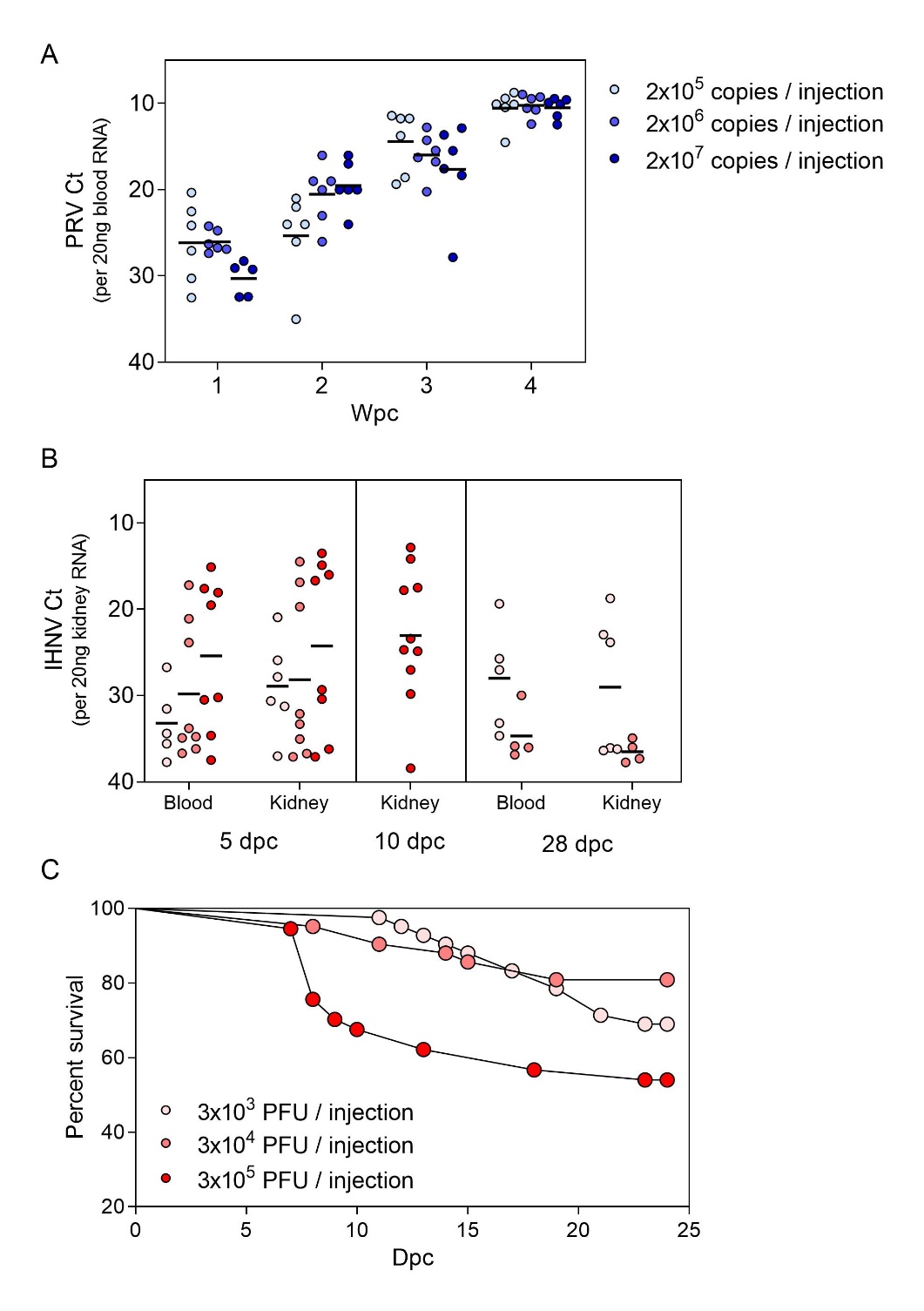


**Fig. S6**. IHNV and PRV pilot challenge data. (A) Transcriptional blood load of PRV L1 genomic segments presented as qPCR threshold cycle (Ct) following intraperitoneal injection of three concentrations of PRV inoculate. Unculturable inoculating material presented as copies of PRV L1 segments per injection as determined by qPCR (B) Transcriptional blood and/or kidney load of IHNV genomic material presented as qPCR threshold cycle (Ct) following intraperitoneal injection of three concentrations of IHNV. Inoculating material concentration presented as viable viral plaque forming units (PFU) per injection. (C) Kaplin-Meier survival curves of fish populations at three doses of IHNV intraperitoneal injection.
